# Corneal Allografts: Factors for and against Acceptance

**DOI:** 10.1155/2021/5372090

**Published:** 2021-10-03

**Authors:** Justyna Sakowska, Paulina Glasner, Maciej Zieliński, Piotr Trzonkowski, Leopold Glasner

**Affiliations:** ^1^Department of Medical Immunology, Medical University of Gdańsk, Dębinki 7 Street, Building 27, 80-211 Gdańsk, Poland; ^2^Department of Anaesthesiology and Intensive Care, Medical University of Gdańsk, Mariana Smoluchowskiego 17 Street, 80-214 Gdańsk, Poland; ^3^Department of Ophthalmology, Medical University of Gdańsk, Mariana Smoluchowskiego 17 Street, 80-214 Gdańsk, Poland

## Abstract

Cornea is one of the most commonly transplanted tissues worldwide. However, it is usually omitted in the field of transplantology. Transplantation of the cornea is performed to treat many ocular diseases. It restores eyesight significantly improving the quality of life. Advancements in banking of explanted corneas and progressive surgical techniques increased availability and outcomes of transplantation. Despite the vast growth in the field of transplantation laboratory testing, standards for corneal transplantation still do not include HLA typing or alloantibody detection. This standard practice is based on immune privilege dogma that accounts for high success rates of corneal transplantation. However, the increasing need for retransplantation in high-risk patients with markedly higher risk of rejection causes ophthalmology transplantation centers to reevaluate their standard algorithms. In this review we discuss immune privilege mechanisms influencing the allograft acceptance and factors disrupting the natural immunosuppressive environment of the eye. Current developments in testing and immunosuppressive treatments (including cell therapies), when applied in corneal transplantation, may give very good results, decrease the possibility of rejection, and reduce the need for retransplantation, which is fairly frequent nowadays.

## 1. Introduction

Corneal transplantation (keratoplasty) is a common procedure performed in the treatment of many vision-impairing diseases. In most cases, it is conducted due to optical reasons (loss of vision) or due to tectonic reasons (restoring damaged cornea surface). Penetrating keratoplasty in which full-thickness of the cornea is transplanted is the most common procedure. However, lamellar keratoplasties in which selected layers of the cornea are transplanted have recently gained significance in clinical setting. Corneal transplantation is the most successful and the most frequently performed solid organ transplantation with 185000 procedures conducted per year worldwide [1]. Corneal graft survival is as high as 90% in low-risk patients [2] with only topical use of immunosuppressants. Unfortunately, rejection rates of corneal grafts in patients qualified as high-risk are similar to kidney or heart transplants, and the use of immunosuppressants topically and systemically is often inadequate [3]. Such discrepancy in survival rates is due to the fact that cornea is an immune-privileged site, and there are specific conditions that disrupt this privilege.

**Figure 1:**
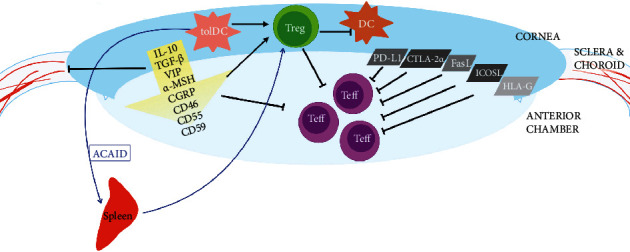
Mechanisms of immune privilege in the eye. tolDC: tolerogenic dendritic cell; Treg: T regulatory cell; DC: dendritic cell; Teff: effector T cell; IL-10: interleukin 10; TGF-*β*: transforming growth factor *β*; VIP: vasoactive intestinal peptide; *α*-MSH: *α* melanocyte-stimulating hormone; CGRP: calcitonin gene-related peptide; PD-L1: programmed death ligand 1; CTLA-2*α*: cytotoxic T lymphocyte-associated antigen-2*α*; FasL: Fas ligand; ICOSL: inducible costimulatory molecule ligand; HLA-G: human leukocyte antigen G; ACAID: anterior chamber-associated immune deviation.

Immune privilege is a set of characteristics and mechanisms that together create an immunosuppressive microenvironment in the cornea and anterior chamber of the eye. The key points of immune privilege summarized in Figure 1 are (1) blood-ocular barrier, (2) immunosuppressive environment of the aqueous humor (AqH), (3) expression of Fas cell surface death receptor ligand (FasL) and programmed death receptor 1 ligand (PD-L1) on corneal and iris cells, and (4) anterior chamber-associated immune deviation (ACAID) and the presence of regulatory T cells (Tregs) [3].

## 2. Immune Privilege of the Eye

### 2.1. Vascular Privilege

The cornea is the central surface part of the eye, and it must be clear to perform its function. In physiological conditions, it remains avascular. This characteristic contributes to its transparency and constitutes the mechanism of immune privilege simultaneously. Cornea lacks both blood vessels and lymphatics, thanks to many antiangiogenic factors [4]. Thrombospondin 1 (TSP-1), endostatin, and pigment epithelium-derived factor (PEDF) were all found both in corneal tissue and AqH, and it was proven that they inhibit blood vessel formation [5–7]. Another set of soluble factors, vasoactive intestinal peptide (VIP), *α*-melanocyte-stimulating hormone (*α*-MSH), transforming growth factor *β* (TGF-*β*), was shown to inhibit lymph vessel formation [8, 9]. Vascular endothelial growth factor C (VEGF-C), which binds to VEGFR-3 receptor expressed by lymphatic endothelial cells, promotes generation of these cells and induces angiogenic response. However, when VEGFR-3 is expressed on corneal epithelial cells, it binds to VEGF-C and limits its availability contributing to the antiangiogenic environment. Similar competitive mechanism of action is presented by soluble VEGFR-1 [10]. Alternative splicing of VEGFR-2 genes results in formation of a soluble VEGFR-2 that was shown to inhibit infiltration of both types of vessels into the cornea [11]. Tumor necrosis factor-related apoptosis-inducing ligand (TRAIL) and tissue plasminogen activator (tPA) expressed on vascular endothelial cells are also proangiogenic. Nevertheless, in the presence of low serum levels in AqH, they were found to induce apoptosis of endothelial cells [12]. This finely tuned system may be disrupted in the course of some ocular diseases or any kind of stimulation such as trauma, infection, or corneal transplantation and then induce invasion of conjunctival vessels into the cornea.

### 2.2. Soluble Immunosuppressive Molecules

The immunosuppressive properties of anterior chamber fluid were identified over 30 years ago [13], and since then, many studies have contributed to elucidating specific factors playing a role in this phenomenon. The AqH is generated by ciliary epithelium and retinal pigment epithelial cells (RPE) [14]. The new 3-compartment model of blood-aqueous barrier describes that the AqH fluid is diffused by ciliary cells, and it is protein-free. The low levels of plasma proteins detected come from the iris stroma, where they are stored at concentrations higher than in AqH [15]. Additionally, the fluid is rich in immunomodulatory molecules and cytokines (Table 1). TGF-*β* is a well-known immunosuppressive cytokine and was shown to suppress interferon *γ* (IFN-*γ*) production and induce TGF-*β* production by T effector cells [16]. In mouse models of ACAID, it was shown to be indispensable in generation of the tolerogenic phenotype of F4/80+ antigen-presenting macrophages in the cornea. TGF-*β* increases expression of F4/80 and CD1d while downregulating expression of costimulatory molecules [17]. Together with *α*-MSH, it induces T regulatory cells and inhibits the production of Th1 cytokines, as well as suppresses the activity of macrophages, dendritic cells (DCs), and neutrophils [16–18]. *α*-MSH is produced by RPE, and its expression can be upregulated in an autocrine fashion as well as induced in macrophages [18]. Other soluble factors that maintain the immunosuppressive environment of the AqH include interleukin 10 (IL-10), PEDF [19], calcitonin gene-related peptide (CGRP) [20, 21], complement regulatory proteins (CD59, membrane cofactor protein (MCP, CD46), decay accelerating factor (DAF, CD55)) [22, 23], migration inhibitory factor (MIF) [24], neuropeptide Y (NPY), somatostatin (SOM) [25], and VIP [18, 26].

### 2.3. Membrane-Bound Immunosuppressive Molecules

Antigen-presenting cells (APCs) in healthy cornea can be in immature state or, as mentioned above, they can present tolerogenic phenotype recognized as lower expression of MHC class II and costimulatory molecules [18]. Moreover, epithelial cells of the cornea express nonclassical MHC class I molecules such as HLA-G [27], which constitutes the mechanism of immune response evasion and inhibition towards effector T cells and NK cells [28]. Many structures of the eye, including cornea, express the following immunomodulatory molecules: FasL [29, 30], PD-L1 [31], GITRL [32], ICOSL [33], galectin-9 (Gal-9) [34], B7-H3 [3], CTLA-2*α* [35, 36], and membrane-bound complement regulatory proteins (CD59, CD55, and CD46) [22] (Table 1).

FasL, PD-L1, and Gal-9 are ligands of inhibitory immune checkpoints and their interaction with T cell receptors: Fas (CD95), PD-1, and Tim-3, respectively, induce apoptosis of activated T cells. FasL and PD-L1 suppress proliferation of T cells. Additionally, PD-L1 suppresses early activation of T cells and cytokine production [37]. The importance of FasL and PD-L1 expression in the allograft acceptance was presented in mouse models [29, 31, 38, 39]. Novel immune checkpoint V-domain immunoglobulin suppressor of T cell activation (VISTA) is both ligand and receptor that shows structural similarities to PD-1 and PD-L1 [40, 41]. It is expressed on APCs and T cells, and it was proven to suppress T cell proliferation and cytokine production *in vitro* [41]. A recent study reported VISTA expression on CD11b+ cells in corneal stroma and its possible role in the acceptance of corneal allografts in mice [42].

Ligands of glucocorticoid-induced tumour necrosis factor receptor family-related protein (GITR) (GITRL) and inducible costimulatory molecule (ICOS) (ICOSL) play a role in peripheral tolerance by inducing regulatory phenotype of effector T cells. Blocking these receptors in mouse models of transplantation increased graft rejection rates [32, 33, 43]. Cytotoxic T lymphocyte-associated antigen-2*α* (CTLA-2*α*) expressed on corneal endothelium is another molecule capable of generating T regulatory cells and contributing to suppression of T cells activation [36, 44].

### 2.4. Anterior Chamber-Associated Immune Deviation and Regulatory T Cells

The immunosuppressive milieu is necessary for proper functioning of ACAID—a mechanism of the cornea that prevents development of delayed-type hypersensitivity (DTH) in response to antigens. DTH starts with recognizing a foreign antigen and presenting it by corneal APCs to T cells. Activated T cells differentiate into Th1 cells producing predominantly IFN-*γ*. DTH-induced inflammation can lead to cell death, tissue remodeling, and fibrosis. Due to limited regenerative potential of the cornea, it can cause vision impairment [16].

ACAID, as a part of immune privilege, is dependent on the immunosuppressive environment of the eye that ensures differentiation of tolerogenic APCs in the eye. It is an antigen-specific response, in which sensitized F4/80+ cells migrate through the bloodstream preferably to the spleen, where they induce differentiation of tolerogenic B cells. Then, B cells act as antigen-presenting cells and generate antigen-specific Tregs [51, 52]. Additionally, NKT cells, IL-10-producing T cells, and *γ*/*δ* T cells take part in ACAID induction. The effects of ACAID are (1) inhibition of Th1 response development mediated by CD4+CD25+ Tregs, (2) suppression of already formed Th1 efferent response mediated by CD8+CD103+ regulatory cells, and (3) modulation of isotype switching in B cells toward noncomplement-binding antibodies [3].

T regulatory cells, defined by expression of Foxp3 transcription factor both ACAID generated and induced by the corneal immunosuppressive microenvironment, contribute to preventing inflammation in the eye [35]. There are many mechanisms by which Tregs act upon T effector cells and APCs. Tregs secrete immunosuppressive cytokines: IL-10, IL-35, and TGF-*β* which inhibit the activity of effector cells and induce tolerogenic phenotype of T cells and APCs. Another characteristic of Tregs is high expression of IL-2 receptor—CD25—that strongly binds to IL-2 and deprives other effector cells of this interleukin that is essential for activation. By expressing CD39 and CD73, Tregs catalyze dephosphorylation of extracellular ATP to adenosine, which suppresses effector T cell function through adenosine receptor 2A. Tregs also directly kill the effector cells through perforin and granzyme A and B cytolysis. One of the most significant modes of action is preventing activation of naive T cells by APCs. Tregs express CTLA-4 and LAG3 that block stimulatory molecules on APCs, CD80/86, and MHC class II. These molecules induce inhibitory pathways that lead to suppressing maturation and costimulatory activity of APCs [53]. High expression of other inhibitory immune checkpoints, such as PD-1, Tim-3, VISTA, GITR, and T cell immunoreceptor with Ig and ITIM domains (TIGIT), also contributes to immunosuppressive capability of Tregs against effector cells [54, 55].

T regulatory cells are a heterogeneous population of cells. Fundamental division of Tregs is based on their origin: thymus-derived Tregs (tTregs) and peripherally induced Tregs (pTregs) [54]. tTregs can be characterized by expression of nuclear transcription factor Helios and surface antigen neuropilin-1 (NRP1) [56]. Interestingly, although the development of autoimmune inflammation in the eye is guarded by tTregs, analogically to type 1 diabetes, thyroiditis, and others, these cells do not take part in the mechanism establishing ACAID [57].

The importance of Tregs in maintaining the immune privilege and preventing autoimmune diseases was shown in studies on murine models of uveitis and dry eye disease [35]. In case of these ocular conditions, pTregs were the prime concern. In the model of dry eye disease, pTregs were reported to degrade to exTregs—lymphocytes secreting IL-17 and INF-*γ* [58, 59]. Similarly, studies on a murine model of corneal transplantation demonstrated the role of Tregs in allograft survival and induction of allotolerance [58, 60, 61]. For example, in high-risk corneal transplants, pTregs show decreased secretion of IL-10 and TGF-*β* immunosuppressive cytokines and lower expression of CTLA-4 [62].

## 3. Rejection Process of Corneal Allografts

### 3.1. Types of Corneal Allograft Rejection

Depending on the part of corneal transplant that is rejected first, we can distinguish four types of rejection.

The most common type is endothelial rejection, which is present in up to 40% of patients with this problem. Inflammatory cells accumulate in the endothelium forming a Khodadoust line, which goes from the periphery of the graft to its central part and causes death of its cells. It is the most severe type of rejection, which usually leads to graft failure [63]. In addition, there is also an inflammation in the anterior chamber. Emerging corneal oedema causes loss of its function. The patient's eye is irritated and shows limbal injection, photophobia, halo rings, and foggy vision.

Subepithelial infiltrates, the second most common type of rejection, may look similar to adenoviral keratitis. This type of rejection can be treated without severe consequences. However, when missed in rough slit lamp examination, it may progress to endothelial rejection [64].

Epithelial rejection is less common. In this type of rejection, lymphocytes accumulate at the donor epithelium. Although this condition does not usually cause significant vision deterioration, it can be the first sign of endothelium rejection. In this case, rejection line can be easily seen with fluorescein staining.

Stromal rejection is the least common type; however, it can accompany neovascularization [65] or even stroma necrosis.

Treatment of all types of rejection is similar. Steroid eye drops (prednisolone 1% or dexamethasone 0.1%) are applied even every 15 minutes. In more severe cases, steroids can be administered in sub-Tenon's injection. Finally, patients may also need systemic corticosteroids (intravenously or orally) in the most difficult cases [66].

### 3.2. Immunopathology of Rejection

Immunological rejection is the most common cause of corneal graft failure. Rejection events were reported in 23% of corneal transplantations; 37% of which ended in graft failure during 5 years of follow-up [67]. They can be diagnosed at least 2 weeks after the transplantation procedure, within which the cornea was clear. However, the immunological rejection usually occurs during the first year posttransplant. The major risk factors for rejection are neovascularization, eye infection, previous transplantation, and, interestingly, younger recipient age.  Table 2 summarizes factors contributing to graft rejection and factors supporting graft acceptance.

Neovascularization is a major risk factor of graft rejection as it provides an easy connection between cornea and lymph nodes for immune cells. Lymphatic vessels create an afferent route for APCs to transport alloantigens to nearby draining lymph nodes (DLNs), where from blood vessels transport alloantigen-specific T effector cells [68, 69]. Both indirect and direct route of alloimmunization occurs during corneal rejection. APCs migrate to conjunctiva-associated lymphoid tissue as well as face and neck lymph nodes, where sensitization of naive T cells takes place. This stands in contrast to generating ACAID-mediated T regulatory cells in the spleen. Activated effector cells migrate back to the cornea and instigate immune response mediated mostly by CD4+ T cells with high INF-*γ* production [70]. It creates a proinflammatory environment promoting activity of the effector cells simultaneously impairing Tregs' suppressive abilities [71]. Preexisting inflammatory conditions in the cornea are another factor increasing risk of graft rejection. Under these conditions, levels of inflammatory cytokines secreted by not only immune cells but also fibroblasts in corneal stroma disrupt the endothelial and smooth muscle cells' attachments at the cornea-conjunctiva border leading to invasion of vessels into the cornea structure. Additionally, proinflammatory environment promotes immune cell activation and impairs Tregs functioning. On the other hand, low-risk beds are those with no signs of inflammation and lack of blood and lymph vessels [3].

Other types of cells, including macrophages, NK cells, and granulocytes, are also present at the site of rejection. Specific interactions between these immune cells and corneal tissue are yet to be described in more detail [72].

## 4. Future Directions for Improving Corneal Transplantation Outcomes

### 4.1. HLA Matching

Crucial element of most transplantations is donor-recipient HLA matching. However, it is not typically performed in corneal allotransplants. There are two reasons that supported establishing this practice in the clinic. First of all, the immune privilege of the cornea greatly contributes to high graft survival rates, although in high-risk patients and those undergoing repeated transplantations, it is severely compromised. Secondly, early studies on HLA matching in corneal transplantation showed contradictory results concerning prolongation of grafts survival [73–75]. These studies were later disproved with development of more precise typing methods based on molecular biology, as opposed to the serological technique used in the first studies. Retrospective assessment of samples from collaborative corneal transplantation studies (CCTS) showed erroneous HLA typing, especially of HLA-DR antigens [76]. Current research indicates benefits from HLA class I typing. The results presented correlation of increased rejection rate with higher number of mismatches [77–80]. HLA class II matching, however, did not prove to be beneficial [81]. Another standard testing before transplantation is detection of recipient alloantibodies and their specificity. Matching them with donor HLA databases (virtual PRA (vPRA)) or donor HLA antigens (virtual crossmatch (V-XM)) better predicts patients' transplantability [82, 83]. Novel concept of HLA matching is based on specific immunogenic epitopes of HLA antigens termed eplets. It enables to predict alloimmunity development in recipients negative for donor-specific antibodies (DSA) [84]. These are routine practices for most solid organ transplants. When implemented in corneal transplantation, they may improve graft survival rates, especially in high-risk cases [75].

### 4.2. Rejection Markers

The rejection process might be dependent on many interconnected factors that result in breaking the ocular immune privilege and graft failure which is observed in the opacity of the cornea. Currently, studies focus on finding early predictors of graft rejection that would allow for rapid treatment and prolong the graft survival. Some of the proposed markers include soluble factors (cytokines, chemokines, and proangiogenic factors) or cellular characteristics (immune cells density, expression of adhesion and costimulatory molecules, APCs migration and activation, and endothelial cell density) [85]. Case studies involving *in vivo* confocal microscopy revealed increased number of activated keratocytes [86] and cells that have dendritic-like morphology along with altered epithelial cells [87]. In corneal rejection setting, VEGF-C was shown to be highly upregulated. As a proangiogenic factor, it not only increased vascularization but also stimulated maturation of APCs, which might have contributed to more efficient allosensitization following the transplantation [88]. Flynn et al. were able to obtain AqH from patients who rejected corneal transplant and analyze it for quantity of cells and cytokines. They observed a prominent presence of CD14+ leukocytes indicating the important role of APCs in rejection. IL-6 proinflammatory cytokine, CXCL10, CCL2, and CCL3 chemokines, and eotaxin were elevated during the rejection incident. Importantly, aspiration of AqH performed during active rejection process did not present any complications. Therefore, this procedure could become a valid diagnostic tool [89].

### 4.3. Inhibition of Neovascularization

One of the researched approaches of manipulating defective immune privilege is targeting the vascular system. Following the process of immune response to alloantigen blocking either efferent lymph vessels or afferent blood vessels may improve allograft outcomes, although transport of sensitized APCs to neighboring lymph nodes seems to play a crucial role [90, 91]. A molecular trap designed to neutralize VEGF-A and tested in mouse model of corneal transplantation was shown to effectively inhibit angiogenesis and lymphangiogenesis and therefore significantly improve graft survival. Further, the blocking resulted in decreased migration of macrophages to the transplanted tissue [92, 93]. Human clinical trials focus on an already approved cancer drug, bevacizumab (an anti-VEGF-A monoclonal antibody), and its use in corneal conditions. Topical application resulted in reduction of vessel diameter and seems to be a relatively safe treatment for corneal neovascularization [94, 95]. Another tested anti-VEGF antibody, ranibizumab, successfully reduced vascularization in examined animals [96]. However, in humans, it performed worse compared to bevacizumab [97].

### 4.4. Inhibition of Cell Migration

Cytokines and chemokines upregulated at the site of inflammation lead to intensified migration and infiltration of leukocytes. Vascularized and inflamed cornea presents altered chemokine expression that promotes migration of cells. In a high-risk mouse model, Amescua et al. observed the key role of CXCL1 in corneal tissue, which is later followed by upregulation of CXCL9 and CXCL10 [98]. The significance of these chemotactic factors, as well as CCL5, in graft rejection arose from promoting T cell infiltration to transplanted cornea. Moreover, these studies indicated therapeutic potential of blocking CXCL1 and chemokine receptors CCR5 and CXCR3 that were shown to decrease allograft rejection incidence [69, 98]. CCR1 receptor shares its ligand, CCL5, with CCR5. Therefore, it was also implicated in corneal graft rejection. CCR1 knock-out mouse model showed increased allograft survival accompanied by decrease in leukocyte migration to the cornea [99].

Hua et al. demonstrated that inflamed host beds present high risk of rejection by supporting maturation and migration of APCs to DLNs. Both graft- and host-derived mature CCR7+ APCs were recruited to DLNs, in which CCL9 and CCL21 were increased in the case of inflammation. Importantly, migration of APCs was inhibited by anti-CCL9 and anti-CCL21 in an *in vitro* test, therefore, indicating new targets for drug development [100]. Another target could be a preoperative manipulation of corneal tissue by incubation with IL-10 and TGF-*β*, which was shown to alter maturation of residing donor APCs toward tolerogenic phenotype. The presence of tolerogenic APCs significantly decreased allosensitization of CD4+ T cells and their infiltration into grafted tissue, thus prolonging the graft survival [101].

### 4.5. Cell Therapy with T Regulatory Cells

Harnessing the potential of regulatory T cells is one of the main directions in developing therapies for rejection of various types of grafts and autoimmune diseases [54].

Studies on mouse model of corneal transplantation presented a significant decrease in graft rejection in high-risk beds after adoptive transfer of allosensitized T regulatory cells. Chauhan et al. observed differences in Foxp3 expression in DLNs between graft rejectors and acceptors. Allospecific Tregs isolated from acceptors were the most potent suppressors of activated T cells proliferation. They were even more potent than Tregs isolated from naive mice. Subsequently, intravenous adoptive transfer of these allospecific Tregs improved graft survival, contrary to Tregs from rejectors and naive mice. No differences in Tregs level were observed at the site of rejection between acceptors and rejectors. Therefore, the authors suggested superior role of Tregs in suppressing antigen presentation than in peripheral regulation of activated T cells [61]. However, a group led by Inomata observed, in addition to decrease in Foxp3 expression, lower frequency of pTregs in DLNs in rejectors. pTregs isolated from low-risk recipients presented better suppressive capacity. Additionally, upon adoptive transfer to high-risk mice these pTregs reduced rejection incidence to a level seen in low-risk mice [62].

Interestingly, Coco et al. demonstrated a protective role of Tregs directly on epithelial cells of the cornea. Combination of mouse model of corneal transplantation and *in vitro* experiments imitating proinflammatory environment of the rejection process showed superior capacity of acceptors' Tregs to produce IL-10 [102].

As the first experiments of cell therapy with ex vivo expanded Tregs were safely implemented in type 1 diabetes mellitus and graft versus host disease (GvHD) in humans [103, 104], it might be the future direction in corneal graft rejection therapy as well. Such cell therapy with polyclonal *in vitro*-induced Tregs administered intravenously was proven to be successful in limiting rejection risk of fully mismatched corneal allografts in mice [105]. The study by Inomata et al. assessed adoptive transfer of tTregs, which resulted in moderate graft survival improvement [62]. The fairly easy access to transplantation site could be used for targeted administration of Tregs, as demonstrated by Shao et al. Naive Tregs injected subconjunctivally inhibited maturation of APCs and their migration to DLNs and increased concentration of anti-inflammatory cytokines, therefore, resulting in improved graft survival [106]. Following the reports on superior suppressive quality of antigen-specific Tregs, they became the recent focus of cell therapy [107]. It could also be the case for corneal transplantation, as mentioned above [61]. Unfortunately, generating antigen-specific Tregs proves to be challenging [107].

Another therapeutic possibility is improving the immunosuppressive potential of T regulatory cells *in vivo* with low-dose IL-2. It promotes generation of Tregs able to suppress T effector cells through upregulation of high Foxp3 expression and STAT5-dependent production of IL-10 and TGF-*β*. It has already been tested in a mouse model of corneal transplantation with positive results [108].

### 4.6. Immune Checkpoints and Costimulatory Receptor Modulation

Inhibitory immune checkpoints play a significant role in ensuring immune privilege of the eye. Taking advantage of this fact could be the new route in corneal rejection immunotherapy. So far, these checkpoints have been tested predominantly in animal models of corneal transplantation. Early experiments conducted by Hoffmann et al. reported improved graft survival with systemic use of CTLA-4-Ig fusion protein, although topical use seemed to worsen the outcome [109]. A clever approach was to subject the graft to CTLA-4-Ig prior to transplantation, which presented an advantage of eliminating potential side effects of systemic treatment. Such manipulation improved allograft survival in vascularized host beds. Moreover, additional UV-B irradiation of the graft gave even better results [110]. Similarly, Watson et al. used PD-L1-Ig fusion protein that prolonged survival of the corneal grafts [111].

The opposing approach involves blocking costimulatory molecules. Blocking antigen presentation with anti-CD80/86 antibodies reduced allograft rejection rates, which was not surprising [112]. Treatment with monoclonal antibody against Tim-1, a stimulatory molecule present on activated T cells, could be very promising. In addition to decreased level of activated T cells, an elevated proportion of Tregs was reported. A reversal of proinflammatory cytokine milieu induced by transplantation was also observed. All of these resulted in improved graft survival [113]. Although the costimulatory ICOS/ICOSL pathway seems to work differently in the cornea and rather promote tolerance, neither anti-ICOS nor anti-ICOSL antibodies had any influence on graft survival time [33, 111].

## 5. Conclusions

The knowledge of corneal microenvironment, immune privilege, graft rejection, and allotolerance accumulated over the years is vast. However, the majority of studies is based on murine models. Therefore, there is a need for researching these concepts in humans, especially the concepts concerning rejection and tolerance of corneal allografts. Despite the relatively good outcomes of corneal transplantation, an increasing number of high-risk patients poses the need for improvements in testing and treatment for rejection. It could be beneficial in lowering costs and reducing the necessity of repeating transplantation procedures. The constantly expanding portfolio of possible immunomodulatory therapies, some of which are already approved or under human clinical trials (bevacizumab, ex vivo-expanded Tregs, and CTLA-4-Ig), could be the future of treatment in corneal transplantation.

## Figures and Tables

**Table 1:** Soluble and cell surface factors providing immune privilege of the eye.

Factor	Source	Target cells	Mechanism
Soluble:			
TGF-*β*	Tregs Epithelium [45]	DCs T effector cells	Induction of tolerogenic phenotype of DCs Suppression of IFN-*γ* production by effector T cell Production of TGF-*β* by effector T cells
*α*-MSH	RPE Macrophages	Tregs T effector cells Macrophages DCs Neutrophils	Induction of Tregs Suppression of Th1 cytokines production Induction of IL-10 production by macrophages
IL-10	Tregs M2 macrophages RPE	DCs T effector cells	Inhibition of IL-12 production by macrophages
PEDF	RPE Iris-ciliary body Cornea Retina	Macrophages	Induction of IL-10 production by macrophages Inhibition of NO production by macrophages
SOM	Epithelium Endothelium Iris-ciliary body Retina [46]	T cells	Suppression of IFN-*γ* production by effector T cells Stimulation of TGF-*β* production Induction of Tregs Induction of *α*-MSH production
CGRP	Terminal sensory nerves in choroid	Macrophages	Suppression of TNF-*α* production Suppression of antigen presentation
CD46, CD55, CD59	Epithelium Stroma AqH	Complement proteins	Interfering with membrane attack complex building
MIF	Endothelium Keratocytes Immune cells	NK cells	Inhibition of perforin release
NPY	Inner nuclear and ganglion cell layers	Macrophages	Induction of coexpression of Arginase1 and NOS2 in resting macrophages to act as suppressor cells
VIP	Iris-ciliary body [47]	Macrophages Lymphocytes Endothelium	Increasing expression of anti-inflammatory Toll-like receptors
Membrane-bound:			
HLA-G	Epithelium Stroma Endothelium	NK cells T cells	Inhibition of lytic activity of NK cells and cytotoxic T cells Shifting T cells to Th2 phenotype Inhibition of T cells' proliferation Induction of Treg cells and tolDCs [48]
FasL	Epithelium [49] Endothelium [50] Retina Iris-ciliary body	Activated T cells APCs	Apoptosis of Fas+ cells
PD-L1	Endothelium Stroma Iris-ciliary body	CD4+ T cells CD8+ T cells	Apoptosis of PD-1+ cells Inhibition of proliferation and IFN-*γ* production by Th1 cells [44]
GITRL	Endothelium Iris-ciliary body Retina	Tregs	Expansion of Tregs in corneal tissue Suppressing T effector cells
ICOSL	Cornea Iris-ciliary body Retina	Tregs	Induction of Tregs Suppressing T effector cells Involvement in ACAID
Gal-9	Epithelium Endothelium Iris-ciliary body Retina	Tregs	Promotes Tregs activity through Tim-3
B7-H3	Endothelium Iris-ciliary body		Induction of ACAID tolerance
CTLA-2*α*	RPE	Effector T cells	Induction of pTregs Stimulation of TGF-*β* production

**Table 2:** Factors influencing corneal graft acceptance and rejection. ACAID: anterior chamber-associated immune deviation; HLA: human leukocyte antigens.

Factors for corneal graft tolerance	Factors for corneal graft rejection
Avascularity	Neovascularization
Immune privilege: immunosuppressive microenvironment, ACAID	Inflammation of the eye: autoimmune or infectious
Histocompatibility	HLA mismatches
First corneal transplantation	Previous corneal transplantation

## Abbreviations

AqH: Aqueous humor
FasL: Fas cell surface death receptor ligand
PD-L1: Programmed death receptor 1 ligand
ACAID: Anterior chamber-associated immune deviation
Tregs: Regulatory T cells
APCs: Antigen-presenting cells
TGF-*β*: Transforming growth factor *β*
IFN-*γ*: Interferon *γ*
IL-10: Interleukin 10
DTH: Delayed-type hypersensitivity
MHC: Major histocompatibility complex
HLA: Human leukocyte antigens
DLNs: Draining lymph nodes.

## References

[1] Gain P., Jullienne R., He Z. (2016). Global survey of corneal transplantation and eye banking. *JAMA Ophthalmology*.

[2] Amouzegar A., Chauhan S. K. (2017). Effector and regulatory T cell trafficking in corneal allograft rejection. *Mediators of Inflammation*.

[3] Hori J., Yamaguchi T., Keino H., Hamrah P., Maruyama K. (2019). Immune privilege in corneal transplantation. *Progress in Retinal and Eye Research*.

[4] Bock F., Maruyama K., Regenfuss B. (2013). Novel anti(lymph)angiogenic treatment strategies for corneal and ocular surface diseases. *Progress in Retinal and Eye Research*.

[5] Sekiyama E., Nakamura T., Cooper L. J. (2006). Unique distribution of thrombospondin-1 in human ocular surface epithelium. *Investigative Ophthalmology & Visual Science*.

[6] Sekiyama E., Nakamura T., Kawasaki S., Sogabe H., Kinoshita S. (2006). Different expression of angiogenesis-related factors between human cultivated corneal and oral epithelial sheets. *Experimental Eye Research*.

[7] Tan Y., Cruz-Guilloty F., Medina-Mendez C. A. (2012). Immunological disruption of antiangiogenic signals by recruited allospecific T cells leads to corneal allograft rejection. *The Journal of Immunology*.

[8] Bock F., Onderka J., Braun G. (2016). Identification of novel endogenous anti(lymph)angiogenic factors in the aqueous humor. *Investigative Ophthalmology & Visual Science*.

[9] Oka M., Iwata C., Suzuki H. I. (2008). Inhibition of endogenous TGF-*β* signaling enhances lymphangiogenesis. *Blood*.

[10] Ambati B. K., Nozaki M., Singh N. (2006). Corneal avascularity is due to soluble VEGF receptor-1. *Nature*.

[11] Albuquerque R. J. C., Hayashi T., Cho W. G. (2009). Alternatively spliced vascular endothelial growth factor receptor-2 is an essential endogenous inhibitor of lymphatic vessel growth. *Nature Medicine*.

[12] Regenfuß B., Dreisow M.-L., Hos D., Masli S., Bock F., Cursiefen C. (2015). The Naïve murine cornea as a model system to identify novel endogenous regulators of Lymphangiogenesis: TRAIL and rtPA. *Lymphatic Research and Biology*.

[13] Kaiser C. J., Ksander B. R., Streilein J. W. (1989). Inhibition of lymphocyte proliferation by aqueous humor. *Regional Immunology*.

[14] Kawanaka N., Taylor A. W. (2011). Localized retinal neuropeptide regulation of macrophage and microglial cell functionality. *Journal of Neuroimmunology*.

[15] Freddo T. F. (2013). A contemporary concept of the blood-aqueous barrier. *Progress in Retinal and Eye Research*.

[16] Taylor A. W. (2007). Ocular immunosuppressive microenvironment. *Chemical Immunology and Allergy*.

[17] Kosiewicz M. M., Alard P. (2004). Tolerogenic antigen-presenting cells: regulation of the immune response by TGF-*β*-treated antigen-presenting cells. *Immunologic Research*.

[18] Taylor A. W., Ng T. F. (2018). Negative regulators that mediate ocular immune privilege. *Journal of Leukocyte Biology*.

[19] Zamiri P., Masli S., Streilein J. W., Taylor A. W. (2006). Pigment epithelial growth factor suppresses inflammation by modulating macrophage activation. *Investigative Ophthalmology & Visual Science*.

[20] Torii H., Hosoi J., Asahina A., Granstein R. D. (1997). Calcitonin gene-related peptide and Langerhans cell function. *The Journal of Investigative Dermatology. Symposium Proceedings*.

[21] Toriyama Y., Iesato Y., Imai A. (2015). Pathophysiological function of endogenous calcitonin gene-related peptide in ocular vascular diseases. *The American Journal of Pathology*.

[22] Sohn J. H., Kaplan H. J., Suk H. J., Bora P. S., Bora N. S. (2000). Chronic low level complement activation within the eye is controlled by intraocular complement regulatory proteins. *Investigative Ophthalmology & Visual Science*.

[23] Bora N. S., Gobleman C. L., Atkinson J. P., Pepose J. S., Kaplan H. J. (1993). Differential expression of the complement regulatory proteins in the human eye. *Investigative Ophthalmology & Visual Science*.

[24] Apte R. S., Sinha D., Mayhew E., Wistow G. J., Niederkorn J. Y. (1998). Cutting edge: role of macrophage migration inhibitory factor in inhibiting NK cell activity and preserving immune privilege. *The Journal of Immunology*.

[25] Taylor A. W., Yee D. G. (2003). Somatostatin is an immunosuppressive factor in aqueous humor. *Investigative Ophthalmology & Visual Science*.

[26] Jiang X., McClellan S. A., Barrett R. P., Zhang Y., Hazlett L. D. (2012). Vasoactive intestinal peptide downregulates proinflammatory TLRs while upregulating anti-inflammatory TLRs in the infected cornea. *Journal of Immunology*.

[27] Le Discorde M., Moreau P., Sabatier P., Legeais J.-M., Carosella E. D. (2003). Expression of HLA-G in human cornea, an immune-privileged tissue. *Human Immunology*.

[28] Riteau B., Rouas-Freiss N., Menier C., Paul P., Dausset J., Carosella E. D. (2001). HLA-G2, -G3, and -G4 isoforms expressed as nonmature cell surface glycoproteins inhibit NK and antigen-specific CTL cytolysis. *Journal of Immunology*.

[29] Stuart P. M., Griffith T. S., Usui N., Pepose J., Yu X., Ferguson T. A. (1997). CD95 ligand (FasL)-induced apoptosis is necessary for corneal allograft survival. *The Journal of Clinical Investigation*.

[30] Gregory-Ksander M., Marshak-Rothstein A. (2021). The FasLane to ocular pathology-metalloproteinase cleavage of membrane-bound FasL determines FasL function. *Journal of Leukocyte Biology*.

[31] Hori J., Wang M., Miyashita M. (2006). B7-H1-induced apoptosis as a mechanism of immune privilege of corneal allografts. *Journal of Immunology*.

[32] Hori J., Taniguchi H., Wang M., Oshima M., Azuma M. (2010). GITR ligand-mediated local expansion of regulatory T cells and immune privilege of corneal allografts. *Investigative Ophthalmology & Visual Science*.

[33] Kunishige T., Taniguchi H., Terada M. (2016). Protective role of ICOS and ICOS ligand in corneal transplantation and in maintenance of immune privilege. *Investigative Ophthalmology & Visual Science*.

[34] Shimmura-Tomita M., Wang M., Taniguchi H., Akiba H., Yagita H., Hori J. (2013). Galectin-9-mediated protection from Allo-specific T cells as a mechanism of immune privilege of corneal allografts. *PLoS One*.

[35] Keino H., Horie S., Sugita S. (2018). Immune privilege and eye-derived T-regulatory cells. *Journal of Immunology Research*.

[36] Sugita S., Yamada Y., Horie S. (2011). Induction of T regulatory cells by cytotoxic T-lymphocyte antigen-2*α* on corneal endothelial cells. *Investigative Ophthalmology & Visual Science*.

[37] Gu Y.-Z., Xue Q., Chen Y.-J. (2013). Different roles of PD-L1 and FasL in immunomodulation mediated by human placenta-derived mesenchymal stem cells. *Human Immunology*.

[38] Osawa H., Maruyama K., Streilein J. W. (2004). CD95 ligand expression on corneal epithelium and endothelium influences the fates of orthotopic and heterotopic corneal allografts in mice. *Investigative Ophthalmology & Visual Science*.

[39] Shen L., Jin Y., Freeman G. J., Sharpe A. H., Dana M. R. (2007). The function of donor versus recipient programmed death-ligand 1 in corneal allograft survival. *Journal of Immunology*.

[40] Flies D. B., Wang S., Xu H., Chen L. (2011). Cutting Edge: A Monoclonal Antibody Specific for the Programmed Death-1 Homolog Prevents Graft-versus-Host Disease in Mouse Models. *The Journal of Immunology*.

[41] Wang L., Rubinstein R., Lines J. L. (2011). VISTA, a novel mouse Ig superfamily ligand that negatively regulates T cell responses. *The Journal of Experimental Medicine*.

[42] Kunishige T., Taniguchi H., Ohno T., Azuma M., Hori J. (2019). VISTA is crucial for corneal allograft survival and maintenance of immune privilege. *Investigative Ophthalmology & Visual Science*.

[43] Bushell A., Wood K. (2007). GITR ligation blocks allograft protection by induced CD25^+^CD4^+^ regulatory T cells without enhancing effector T-cell function. *American Journal of Transplantation*.

[44] Sugita S., Usui Y., Horie S. (2009). Human corneal endothelial cells expressing programmed death-ligand 1 (PD-L1) suppress PD-1+ T helper 1 cells by a contact-dependent mechanism. *Investigative Ophthalmology & Visual Science*.

[45] Tandon A., Tovey J. C. K., Sharma A., Gupta R., Mohan R. R. (2010). Role of transforming growth factor beta in corneal function, biology and pathology. *Current Molecular Medicine*.

[46] Dubovy S. R., Fernandez M. P., Echegaray J. J. (2017). Expression of hypothalamic neurohormones and their receptors in the human eye. *Oncotarget*.

[47] Taylor A. W., Streilein J. W., Cousins S. W. (1994). Immunoreactive vasoactive intestinal peptide contributes to the immunosuppressive activity of normal aqueous humor. *The Journal of Immunology*.

[48] Rizzo R., Bortolotti D., Bolzani S., Fainardi E. (2014). HLA-G molecules in autoimmune diseases and infections. *Frontiers in Immunology*.

[49] Hasby E. A., Saad H. A. (2013). Immunohistochemical expression of Fas ligand (FasL) and neprilysin (neutral endopeptidase/CD10) in keratoconus. *International Ophthalmology*.

[50] Wilson S. E., Li Q., Weng J. (1996). The Fas-Fas ligand system and other modulators of apoptosis in the cornea. *Investigative Ophthalmology & Visual Science*.

[51] Skelsey M. E., Mayhew E., Niederkorn J. Y. (2003). Splenic B cells act as antigen presenting cells for the induction of anterior chamber-associated immune deviation. *Investigative Ophthalmology & Visual Science*.

[52] D’Orazio T. J., Mayhew E., Niederkorn J. Y. (2001). Ocular immune privilege promoted by the presentation of peptide on tolerogenic B cells in the spleen. II. Evidence for presentation by Qa-1. *Journal of Immunology*.

[53] Vignali D. A. A., Collison L. W., Workman C. J. (2008). How regulatory T cells work. *Nature Reviews Immunology*.

[54] Ryba-Stanisławowska M., Sakowska J., Zieliński M., Ławrynowicz U., Trzonkowski P. (2019). Regulatory T cells: the future of autoimmune disease treatment. *Expert Review of Clinical Immunology*.

[55] Zhao H., Liao X., Kang Y. (2017). Tregs: where we are and what comes next?. *Frontiers in Immunology*.

[56] Weiss J. M., Bilate A. M., Gobert M. (2012). Neuropilin 1 is expressed on thymus-derived natural regulatory T cells, but not mucosa-generated induced Foxp3+ T reg cells. *Journal of Experimental Medicine*.

[57] Keino H., Takeuchi M., Kezuka T. (2006). Induction of eye-derived tolerance does not depend on naturally occurring CD4+CD25+ T regulatory cells. *Investigative Ophthalmology & Visual Science*.

[58] Hua J., Inomata T., Chen Y. (2018). Pathological conversion of regulatory T cells is associated with loss of allotolerance. *Scientific Reports*.

[59] Guo J., Zhou X. (2015). Regulatory T cells turn pathogenic. *Cellular & Molecular Immunology*.

[60] Cunnusamy K., Paunicka K., Reyes N., Yang W., Chen P. W., Niederkorn J. Y. (2010). Two different regulatory T cell populations that promote corneal allograft survival. *Investigative Ophthalmology & Visual Science*.

[61] Chauhan S. K., Saban D. R., Lee H. K., Dana R. (2009). Levels of Foxp3 in regulatory T cells reflect their functional status in transplantation. *Journal of Immunology*.

[62] Inomata T., Hua J., Di Zazzo A., Dana R. (2016). Impaired function of peripherally induced regulatory T cells in hosts at high risk of graft rejection. *Scientific Reports*.

[63] Weisenthal R. W., Bouchard C., Rootman D., Tu E., de Freitas D., Weisenthal R. W. (2015). *2015-2016 Basic and clinical science course (BCSC): external disease and cornea section 8*.

[64] Foulks G. N., Krachmer J. H., Mannis M. J., Holland E. J. (2011). Diagnosis and management of corneal allograft rejection. *Cornea 3rd ed Vol 2*.

[65] Mahabadi N., Czyz C. N., Tariq M., Havens S. J. (2021). *Corneal graft rejection. StatPearls*.

[66] Larkin D. F. (1994). Corneal allograft rejection. *The British Journal of Ophthalmology*.

[67] Stulting R. D., Sugar A., Beck R. (2012). Effect of donor and recipient factors on corneal graft rejection. *Cornea*.

[68] Yamagami S., Dana M. R. (2001). The critical role of lymph nodes in corneal alloimmunization and graft rejection. *Investigative Ophthalmology & Visual Science*.

[69] Tan Y., Abdulreda M. H., Cruz-Guilloty F. (2013). Role of T cell recruitment and chemokine-regulated intra-graft T cell motility patterns in corneal allograft rejection. *American Journal of Transplantation*.

[70] Hegde S., Beauregard C., Mayhew E., Niederkorn J. Y. (2005). CD4(+) T-cell-mediated mechanisms of corneal allograft rejection: role of Fas-induced apoptosis. *Transplantation*.

[71] Tahvildari M., Inomata T., Amouzegar A., Dana R. (2018). Regulatory T cell modulation of cytokine and cellular networks in corneal graft rejection. *Current Ophthalmology Reports*.

[72] Coster D. J., Williams K. A. (2005). The impact of corneal allograft rejection on the long-term outcome of corneal transplantation. *American Journal of Ophthalmology*.

[73] The collaborative corneal transplantation studies (CCTS) (1992). The collaborative corneal transplantation studies (CCTS). Effectiveness of histocompatibility matching in high-risk corneal transplantation. *Archives of Ophthalmology*.

[74] Beekhuis W. H., van Rij G., Renardel de Lavalette J. G., Rinkel-van Driel E., Persijn G., D’Amaro J. (1991). Corneal graft survival in HLA-A- and HLA-B-matched transplantations in high-risk cases with retrospective review of HLA-DR compatibility. *Cornea*.

[75] van Essen T. H., Roelen D. L., Williams K. A., Jager M. J. (2015). Matching for human leukocyte antigens (HLA) in corneal transplantation - to do or not to do. *Progress in Retinal and Eye Research*.

[76] Hopkins K. A., Maguire M. G., Fink N. E., Bias W. B. (1992). Reproducibility of HLA-A, B, and DR typing using peripheral blood samples: Results of retyping in the collaborative Corneal transplantation studies. *Human Immunology*.

[77] Bartels M. C., Otten H. G., van Gelderen B. E., Van der Lelij A. (2001). Influence of HLA-A, HLA-B, and HLA-DR matching on rejection of random corneal grafts using corneal tissue for retrospective DNA HLA typing. *The British Journal of Ophthalmology*.

[78] Böhringer D., Daub F., Schwartzkopff J. (2010). Operational post-keratopasty graft tolerance due to differential HLAMatchmaker matching. *Molecular Vision*.

[79] Beekhuis W. H., Bartels M., Doxiadis I. I. N., van Rij G. (2002). Degree of compatibility for HLA-A and -B affects outcome in high-risk corneal transplantation. *Developments in Ophthalmology*.

[80] V??lker-Dieben H. J., Claas F. H., Schreuder G. M. (2000). Beneficial effect of HLA-DR matching on the survival of corneal ALLOGRAFTS1. *Transplantation*.

[81] Armitage W. J., Winton H. L., Jones M. N. A. (2020). Corneal transplant follow-up study II: a randomised trial to determine whether HLA class II matching reduces the risk of allograft rejection in penetrating keratoplasty. *The British Journal of Ophthalmology*.

[82] Süsal C., Morath C. (2015). Virtual PRA replaces traditional PRA: small change but significantly more justice for sensitized patients. *Transplant International*.

[83] Moszkowska G., Zieliński M., Zielińska H. (2018). Evaluation of Pretransplant donor-specific alloantibodies with different crossmatch techniques. *Transplantation Proceedings*.

[84] Lim W. H., Wong G., Heidt S., Claas F. H. J. (2018). Novel aspects of epitope matching and practical application in kidney transplantation. *Kidney International*.

[85] di Zazzo A., Lee S.-M., Sung J. (2020). Variable responses to corneal grafts: insights from immunology and systems biology. *Journal of Clinical Medical Research*.

[86] Kocaba V., Colica C., Rabilloud M., Burillon C. (2015). Predicting corneal graft rejection by confocal microscopy. *Cornea*.

[87] Niederer R. L., Sherwin T., McGhee C. N. J. (2007). In vivo confocal microscopy of subepithelial infiltrates in human corneal transplant rejection. *Cornea*.

[88] Hajrasouliha A. R., Funaki T., Sadrai Z., Hattori T., Chauhan S. K., Dana R. (2012). Vascular endothelial growth factor-C promotes Alloimmunity by amplifying antigen-presenting cell maturation and Lymphangiogenesis. *Investigative Ophthalmology & Visual Science*.

[89] Flynn T. H., Mitchison N. A., Ono S. J., Larkin D. F. P. (2008). Aqueous humor alloreactive cell phenotypes, cytokines and chemokines in corneal allograft rejection. *American Journal of Transplantation*.

[90] Tahvildari M., Amouzegar A., Foulsham W., Dana R. (2018). Therapeutic approaches for induction of tolerance and immune quiescence in corneal allotransplantation. *Cellular and Molecular Life Sciences*.

[91] Dietrich T., Bock F., Yuen D. (2010). Cutting Edge: Lymphatic Vessels, Not Blood Vessels, Primarily Mediate Immune Rejections After Transplantation. *The Journal of Immunology*.

[92] Bachmann B. O., Bock F., Wiegand S. J. (2008). Promotion of graft survival by vascular endothelial growth factor a neutralization after high-risk corneal transplantation. *Archives of Ophthalmology*.

[93] Cursiefen C., Cao J., Chen L. (2004). Inhibition of hemangiogenesis and LymphangiogenesisafterNormal-Risk corneal transplantation by neutralizing VEGF promotes graft survival. *Investigative Ophthalmology & Visual Science*.

[94] Koenig Y., Bock F., Horn F., Kruse F., Straub K., Cursiefen C. (2009). Short- and long-term safety profile and efficacy of topical bevacizumab (Avastin) eye drops against corneal neovascularization. *Graefe's Archive for Clinical and Experimental Ophthalmology*.

[95] Bhatti N., Qidwai U., Hussain M., Kazi A. (2013). Efficacy of sub-conjunctival and topical bevacizumab in high-risk corneal transplant survival. *Journal of Pakistan Medical Association*.

[96] Liarakos V. S., Papaconstantinou D., Vergados I., Douvali M., Theodossiadis P. G. (2014). The effect of subconjunctival ranibizumab on corneal and anterior segment neovascularization: study on an animal model. *European Journal of Ophthalmology*.

[97] Kim J.-H., Seo H.-W., Han H.-C., Lee J.-H., Choi S.-K., Lee D. (2013). The effect of bevacizumab versus ranibizumab in the treatment of corneal neovascularization: a preliminary study. *Korean Journal of Ophthalmology*.

[98] Amescua G., Collings F., Sidani A. (2008). Effect of CXCL-1/KC production in high risk vascularized corneal allografts on T cell recruitment and graft rejection. *Transplantation*.

[99] Hamrah P., Yamagami S., Liu Y. (2007). Deletion of the chemokine receptor CCR1 prolongs corneal allograft survival. *Investigative Ophthalmology & Visual Science*.

[100] Hua J., Stevenson W., Dohlman T. H. (2016). Graft site microenvironment determines dendritic cell trafficking through the CCR7-CCL19/21 Axis. *Investigative Ophthalmology & Visual Science*.

[101] Tahvildari M., Emami-Naeini P., Omoto M., Mashaghi A., Chauhan S. K., Dana R. (2017). Treatment of donor corneal tissue with immunomodulatory cytokines: a novel strategy to promote graft survival in high-risk corneal transplantation. *Scientific Reports*.

[102] Coco G., Foulsham W., Nakao T. (2020). Regulatory T cells promote corneal endothelial cell survival following transplantation via interleukin-10. *American Journal of Transplantation*.

[103] Marek-Trzonkowska N., Myśliwiec M., Dobyszuk A. (2014). Therapy of type 1 diabetes with CD4^+^CD25^high^CD127-regulatory T cells prolongs survival of pancreatic islets -- Results of one year follow-up. *Clinical Immunology*.

[104] Trzonkowski P., Bieniaszewska M., Juścińska J. (2009). First-in-man clinical results of the treatment of patients with graft versus host disease with human ex vivo expanded CD4+CD25+CD127− T regulatory cells. *Clinical Immunology*.

[105] Guo X., Jie Y., Ren D. (2012). In vitro-expanded CD4^+^CD25^high^Foxp3^+^ regulatory T cells controls corneal allograft rejection. *Human Immunology*.

[106] Shao C., Chen Y., Nakao T. (2019). Local delivery of regulatory T cells promotes corneal allograft survival. *Transplantation*.

[107] Selck C., Dominguez-Villar M. (2021). Antigen-specific regulatory T cell therapy in autoimmune diseases and transplantation. *Frontiers in Immunology*.

[108] Tahvildari M., Omoto M., Chen Y. (2016). In vivo expansion of regulatory T cells by low-dose Interleukin-2 treatment increases allograft survival in corneal transplantation. *Transplantation*.

[109] Hoffmann F., Zhang E. P., Pohl T., Kunzendorf U., Wachtlin J., Bulfone-Paus S. (1997). Inhibition of corneal allograft reaction by CTLA4-Ig. *Graefe's Archive for Clinical and Experimental Ophthalmology*.

[110] Gebhardt B. M., Hodkin M., Varnell E. D., Kaufman H. E. (1999). Protection of corneal allografts by CTLA4-Ig. *Cornea*.

[111] Watson M. P., George A. J. T., Larkin D. F. P. (2006). Differential effects of costimulatory pathway modulation on corneal allograft survival. *Investigative Ophthalmology & Visual Science*.

[112] Kagaya F., Hori J., Kamiya K. (2002). Inhibition of murine corneal allograft rejection by treatment with antibodies to CD80 and CD86. *Experimental Eye Research*.

[113] Tan X., Jie Y., Zhang Y., Qin Y., Xu Q., Pan Z. (2014). Tim-1 blockade with RMT1-10 increases T regulatory cells and prolongs the survival of high-risk corneal allografts in mice. *Experimental Eye Research*.

